# Object to Automobile Law

**Published:** 1908-06

**Authors:** 


### OBJECT TO AUTOMOBILE LAW.

A number of prominent physicians of Atlanta are making a strenuous fight against the passage of Councilman Grant's vehicle ordinance which provides that no vehicles be allowed to stop in front of buildings in the congested district. It further requires that an attendant must be left with such vehicles; also that vehicles must stop when a car in front comes to a stand still to let off passengers.

There were present at the meeting of the committee to pass upon the ordinance, Dr. James B. Baird, Dr. Floyd W. McRae, Dr. William B. Armstrong, Dr. Cyrus W. Strickler, Dr. C. E. Murphey and Dr. B. E. Pearce.

Such a law will impose a great hardship upon the physicians who use automobiles in their practice. The doctor is as a rule a careful chauffeur; in fact this fact is so well recognized in certain large cities that on account of the urgency of his mission the physician is not subject to the usual speed limit.

